# Histone supply: a precious commodity for cell identity

**DOI:** 10.1042/BCJ20253163

**Published:** 2025-09-25

**Authors:** Sara Gonske, Thelma M. Escobar, Alejandra Loyola

**Affiliations:** 1 Department of Biochemistry, University of Washington, Seattle, Washington, 98195, U.S.A; 2 The Institute for Stem Cell and Regenerative Medicine, University of Washington, 98195, U.S.A; 3 Centro Científico y Tecnológico de Excelencia Ciencia & Vida, Fundación Ciencia & Vida, Santiago, 8580702, Chile; 4 Facultad de Ciencias, Universidad San Sebastián, Santiago, 7510602, Chile

**Keywords:** epigenetics, histones, gene expression and regulation, post-translational modification

## Abstract

Histones are critical for eukaryotic cell survival, supporting and packaging DNA into chromatin domains that define gene expression and cellular identity. Fundamental to the establishment of these domains is the adequate histone supply that is tightly regulated from the moment histones are transcribed, synthesized, and recycled, to when they are degraded. In this review, we describe and emphasize each step of the histone supply chain and its impact on chromatin structure and cellular identity. Given the robust studies on histones H3 and H4 supply, we primarily discuss these nucleosome components and their variants while briefly touching on H2A and H2B dynamics. We also highlight central proteins that supervise these processes and relay key studies that explore the consequences and clinical impact of limiting or altering the histone supply chain. Together, these insights underscore the importance of histone homeostasis as a critical determinant of genome stability and cell fate.

## Introduction/Background

### Overview of histones

**Figure 1:**
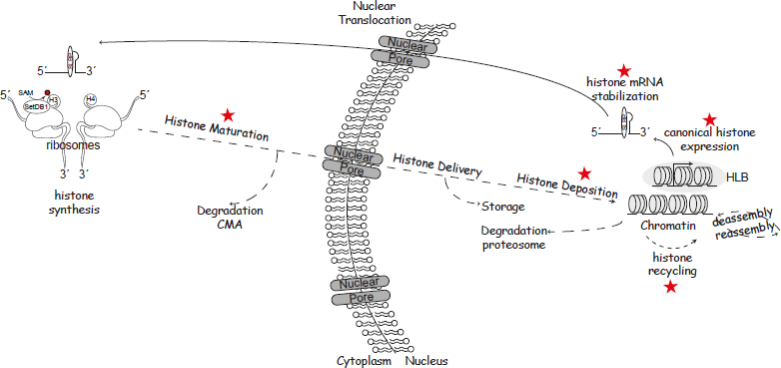
The histone supply chain. The figure illustrates the key processes involved in histone supply, deposition, and maintenance, which are essential for preserving genome integrity and cellular identity. Canonical histone biosynthesis begins with the transcription of histone mRNAs in histone locus bodies (HLBs) during S-phase. These mRNAs, stabilized by the protein SLBP, are transported through the nuclear pore into the cytoplasm, where they are translated on ribosomes. During translation, histone H3 undergoes its first post-translational modification—monomethylation at lysine 9, mediated by SETDB1, which is associated with ribosomes. Newly synthesized histones H3 and H4 then enter a maturation pathway, interacting with various histone chaperones and binding proteins. If this process is disrupted, histones are targeted for degradation through the chaperone-mediated autophagy (CMA) pathway. Properly matured histones are imported into the nucleus via importin-mediated transport, where they engage with nuclear chaperones that facilitate their deposition onto chromatin, forming nucleosomes—the fundamental units of chromatin structure. The histone–DNA association remains dynamic, as nucleosomes are continuously disassembled and reassembled to accommodate DNA-dependent processes. S-phase presents a unique challenge, as chromatin disassembly is required for replication fork progression. During this phase, parental histones are recycled and mixed with newly synthesized histones to maintain chromatin organization. In the nucleus, histone degradation is primarily mediated by the proteasome. The stars in the figure highlight critical regulatory steps where disruptions in histone homeostasis can lead to various diseases, including cancer. SETDB1, SET domain bifurcated histone lysine methyltransferase 1.

As eukaryotic cells and complex organisms arose, so did the need to organize and compact large, intricate genomes where gene expression and cellular identity are safeguarded. Nature’s solution to this challenge yields the histone; a small, positively charged protein, which, when assembled into an octet of core histones and wrapped with 147 base pairs of DNA, forms the functional unit of chromatin, the nucleosome [1]. When incorporated into the genome, nucleosomes lay a blank canvas for the deposition of the ‘histone code’ [2] that directs the packaging of the genome into distinct chromatin domains, dictating gene expression. As gene expression programs define cellular identity, histones prove a precious commodity for the cell. This is demonstrated by the tightly regulated histone supply chain that begins with histone synthesis and ends in histone degradation. Disrupting this chain, including mutations to key chromatin players, is detrimental to the cell and can induce histone scarcity, compromise chromatin structure, and lead to disease (Figure 1). Therefore, proper execution of the histone supply chain and strict regulation of chromatin features, including histone post-translational modifications (PTMs) and histone variants, is imperative to protect genome integrity and cellular identity.

The canonical nucleosome contains two copies of histones H2A, H2B, H3, and H4, each with an outward-facing, N-terminal tail, and a C-terminal tail in the case of H2A, that is subject to various PTMs altering chromatin domains and structure. In addition to these core histones, the linker histone, H1, binds nucleosomes, facilitating higher-order chromatin compaction and organization of the genome. Common PTMs found on histones include phosphorylation, acetylation, methylation, ubiquitination, sumoylation, and ADP-ribosylation on different amino acid residues. Importantly, these PTMs influence the deposition, recognition, spacing, and interactome of the nucleosomes on which they are placed and consequently define the gene expression of associated loci. Specialized proteins known as readers, writers, and erasers are responsible for recognizing, imposing, and removing modifications, respectively [3-5]. Often, ‘a concert’ of proteins regulates a particular PTM within the chromatin. For example, tightly packed heterochromatin*,* marked by di- or tri-methylation of lysine 9 of histone H3 (H3K9me2/3), is read by chromodomain protein heterochromatin protein 1 (HP1), established by SET domain bifurcated histone lysine methyltransferase 1 (SETDB1), G9a-like protein, and suppressor of variegation 3–9 homolog 1/2 (SUV39H1/H2), and removed by PHD (plant homeodomain finger) finger protein 8 [4]. On the other hand, loosely packed euchromatin*,* marked by the methylation of lysine 4 of histone H3 (H3K4me), is read by PHD-domain-containing proteins like bromodomain PHD finger transcription factor (BPTF), established by enzymes including the complex of proteins associated with Set1 (COMPASS) complex, and removed by the lysine-specific histone demethylase 1A (KDM1) and jumonji domain AT-rich interactive domain (ARID)-containing protein 1 families [4]. Indeed, correct ‘orchestrations’ and abundances of these histone modifications and their respective enzyme complexes are extremely important for the maintenance of chromatin structure and gene expression.

Along with PTMs, the existence of histone variants adds complexity to the epigenome. In eukaryotes, an abundant number of histone variants exist for H2A, H2B, and H3. Often, these histone derivatives differ from the canonical analogs by just a few residues in the primary amino acid sequence. Although small, these unique modifications in sequence allow for newfound functions of histone variants, which are critical for marking specific locations throughout the genome. Unlike their canonical equivalents, histone variants are often expressed independently from DNA replication to accommodate dynamic variant demand and supply during the cell cycle and cellular differentiation. To illustrate, in humans, the H3 histone variant centromere protein A (CENP-A) is transcribed during G2/M phase and is incorporated in a distinct region of the chromosome known as the centromere. Deposition of CENP-A defines the centromeric chromatin, which is important for the assembly of the kinetochore machinery and the mitotic spindle [6-9]. Histone variants, their specific functions, and their importance for cellular identity have been well summarized by many groups and should be further reviewed here [10-14].

Integrating the supply, deposition, and maintenance of the individual histone features described above is key to establishing gene expression and cellular identity. Not only must this histone landscape be correctly composed, but it must also be maintained despite several challenges during cellular division and differentiation. Processes including DNA replication, transcription, repair, and recombination disrupt the epigenetic template as parental histones are evicted from their original positions. Key to maintaining or modulating chromatin structure is the redeposition of histones after these processes are completed [15-20]. Importantly, this relies on a tightly regulated cascade of cellular proteins that aid in histone movement and modification, especially histone chaperones which shuttle and direct histones into DNA. These chaperones are also crucial for the incorporation of histone variants at specific genomic locations, both during and outside of S-phase. Thus, the proper assembly of chromatin involves a dynamic interplay between histone supply, variant deposition, and chaperone-mediated trafficking. Still, a critical question in the field remains: how is the chromatin landscape faithfully transmitted to daughter cells to either conserve cellular identity or adapt for cellular differentiation? In this review, we will discuss the role in which histone supply and dynamics contribute to cellular identity and the consequences of when the epigenetic landscape is compromised.

### Overview of histone chaperones: the evolutionary ‘keepers’ of histones and their movement

Histone chaperones have evolved to govern histones and are responsible for histone movement at every point after translation [21]. Although the large, net-positive charge of histones is necessary to bind the negatively charged backbone of DNA, it poses a threat to the cell when left unaccompanied. To overcome this challenge, chaperones bind histones from the moment they are translated to the moment they are deposited into the genome, as well as when they are evicted from chromatin during events such as transcription [14,22-26]. Acidic stretches and intrinsically disordered domains (IDRs) within the histone chaperone help mediate this interaction [27]. Once bound, the histones are passed from one chaperone to the next, ensuring their swift and secure delivery to the DNA by preventing histone aggregation or haphazard interactions.

Unsurprisingly, a diverse family of histone chaperones has co-evolved along with histone variants and PTMs to fill functional niches and maintain the correct epigenetic landscape. Within this family, the chaperones have little structural conservation and have adopted individual mechanisms for moving histones around the cell [26]. For example, the histone chaperone anti-silencing function 1 (ASF1) escorts H3/H4 heterodimers containing almost any flavor of H3 and aids in their transportation from the cytoplasm and their deposition into the DNA [28-30]. On the other hand, more specialized chaperones exist, such as Holliday junction recognition protein (HJURP), a chaperone that only carries H3/H4 heterodimers containing the centromeric variant of H3, CENP-A. HJURP then directs and deposits that variant into the correct location at the centromeres of the chromosome [26,31]. Additional chaperones exist for other H3 variants, including Chromatin assembly factor-1 (CAF-1), which shuttles and deposits H3.1 and H3.2, and Death Domain-Associated Protein (DAXX) and Histone Regulator A (HIRA), which can both transport the variant H3.3 [32-34]. Histone chaperones and their cargo have been reviewed and summarized in detail (Table 1) [26,58,59]. In any case, the discrete structure-guided mechanisms of histone chaperones and the proper supply of histones to feed into them are imperative for the correct movement and placement of histones within chromatin.

**Table 1:** Histone H3 and H4 chaperones

Chaperone	Histone complex	Function	Localization	References
Hsc70	H3 monomer	Prevent misfolding and aggregation	Cytoplasm	[29,35]
Hsp90/Hsp70	H4 monomer	Prevent misfolding and aggregation	Cytoplasm	[35]
PP32/SET	H4 monomer	Inhibits premature acetylation	Cytoplasm	[36]
tNASP/Hsp90	H3-H4	Assembly H3-H4 dimer	Cytoplasm	[29,35]
sNASP	H3-H4	Main cytosolic chaperone. Keeps H3-H4 for nuclear translocation and chromatin deposition	Cytoplasm	[29,35]
HAT1	H3-H4	Acetylates newly synthesized H4	Cytoplasm	[37]
Importins	H3-H4	Nuclear import of H3-H4	Cytoplasm	[38]
ASF1A/B	H3-H4	Transfers H3-H4 to downstream chaperones	Cytoplasm/Nucleus	[39]
RbAp46/48	H3-H4, (H3-H4)_2_	Part of multiple chromatin remodeling complexes	Nucleus	[40]
CAF-1	(H3-H4)_2_	Nucleosome assembly during DNA replication	Nucleus	[41]
HIRA	(H3.3-H4)_2_	Replication-independent nucleosome assembly	Nucleus	[32]
DAXX-ATRX	(H3.3-H4)_2_	H3.3 deposition at telomeres and pericentromeres	Nucleus	[42,43]
DEK	(H3.3-H4)_2_	Chromatin binding and gene regulation	Nucleus	[44]
VPS75	H3-H4	Works with HAT1 for acetylation	Nucleus	[45]
MCM2-DNA polymerase-CTF4	(H3-H4)_2_	H3-H4 deposition to the lagging strand	Nucleus	[46,47]
POLE3-POLE4	(H3-H4)_2_	H3-H4 deposition to the leading strand	Nucleus	[48,49]
TONSL	(H3-H4)_2_	Histone recycling at replication fork	Nucleus	[47]
MCM2	(H3-H4)_2_	Histone recycling at replication fork	Nucleus	[50]
HJURP	CENP-A	CENP-A deposition at centromeres	Nucleus	[51-53]
Mrc1	(H3-H4)_2_	Parental histone segregation	Nucleus	[54,55]
NPM1	(H3-H4)_2_	Histone recycling at replication fork on repressed domains	Nucleus	[56]
Spt6	Octamer	Transcription-coupled nucleosome reassembly	Nucleus	[57]

sNASP, somatic NASP. RbAp, retinoblastoma-associated protein. HAT1, histone acetyltransferase 1. MCM, minichromosome maintenance.

Despite the importance of understanding these structure-guided mechanisms, histone chaperones have proven challenging to study due to their structural and functional diversity. Although united in their chaperoning function, histone chaperones have distinct structural folds and histone binding motifs [26,27]. This means that as biochemists, we must look at each histone chaperone individually to understand which unique parts of their structures adapt them to perform their specific chaperoning role. To make things more complex, chaperones often perform other cellular functions, which can make it difficult to study the isolated chaperoning mechanism. For instance, the histone chaperone nucleophosmin (NPM1) is involved in numerous processes apart from chaperoning, including ribosomal biogenesis and DNA repair [60,61]. Its activity, localization, and interaction network are in part regulated by PTMs added to the chaperone throughout the cell cycle; however, individual PTMs are still being assigned to the specific functions of NPM1 [62]. In the future, it will be important for research groups to continue dissecting the mechanisms of histone chaperones so that we may better understand their individual impact on histone supply, the epigenome, and cellular identity.

## Histone expression and synthesis

### Histone gene expression

The histone supply chain starts with the transcription of the histone genes, divided into two categories: replication-dependent and replication-independent genes (Figure 1). Canonical histones make up the replication-dependent genes and are robustly expressed during S-phase to replenish the histone pool during cellular division. On the other hand, histone variants make up replication-independent genes and are expressed at low levels throughout the cell cycle in a temporal and tissue-dependent manner [63]. Naturally, transcription of these distinct pools must be tightly regulated to maintain a strict balance of these histones and their quantity within the cell at any given moment.

In the human genome, 82 replication-dependent genes are highly expressed during S-phase [64]. Each has been given a unique, identifying gene symbol under the recently adopted ‘Strasbourg nomenclature’ to denote both its encoded protein and phylogenetic context [65]. At the genomic level, this enormous set of genes is partitioned into four clusters named HIST1-HIST4 that are spread across three chromosomes (Table 2). The largest cluster, HIST1, exists on chromosome 6 (HIST1, 6p21-22) and encodes more than 50 genes, while clusters HIST2 and HIST3 are both located on chromosome 1 (HIST2, 1q21 and HIST3, 1q42) and encode 12 and 3 genes, respectively. Finally, HIST4 is found on chromosome 12 (HIST4, 12p12.3) and encodes just one gene. These loci are in syntenic positions in mammals [65], and mouse canonical histone genes are organized into three clusters: HIST1, HIST2, and HIST3, located on mouse chromosomes 13, 3, and 11, respectively [66]. In these metazoans, the high copy number of histone genes compensates for the high demand for canonical histones during cellular division as the histone pool swiftly doubles.

**Table 2:** Histone clusters

Genomic location	Histone encoded	Number of gene copies
Chr 6p22	H2A, H2B, H3, H4	12 H2A genes, 15 H2B genes, 10 H3 genes, 12 H4 genes
Chr 1q21	H2A, H2B, H3, H4	4 H2A genes, 2 H2B genes, 4 H3 genes, 2 H4 genes
Chr 1q42	H3	1 H2A gene, 1 H2B gene, 1 H3 gene
Chr 12q13	H4	1 H4 gene

Expression of these replication-dependent histone clusters is stringently co-ordinated to maintain a constant supply of canonical histones during S-phase. Despite being located on different chromosomes, the canonical histone genes in clusters 1 and 2 condense into histone locus bodies (HLBs) whose composition regulates their transcription throughout the cell cycle [67]. Critically, at the G1/S checkpoint, the cyclin-dependent kinase 2 (E-CDK2) complex phosphorylates and activates the transcription factor nuclear protein ataxia–telangiectasia (NPAT), which in turn, induces the transcription of the replication-dependent histones [68]. Simultaneously, the cyclin E-CDK2 complex initiates DNA replication by promoting origin firing and S-phase entry [69]. The HLB then grows to incorporate transcriptional and processing machineries to support the high-volume production of canonical histone mRNAs. These transcripts are unique among metazoan mRNAs as they do not contain introns, nor are they polyadenylated. Instead, histone mRNAs terminate in a conserved stem-loop structure at their 3’-end, which permits their recognition by the stem-loop binding protein (SLBP). Upon SLBP binding, the U7 small nuclear ribonucleoprotein complex recognizes a downstream 3’ sequence element and directs splicing of the nascent transcript to form a mature histone mRNA [70,71]. SLBP binding is also critical for downstream processes, including mRNA stability, nuclear export, and translation of the encoded histone proteins (Figure 1). At the end of S-phase, histone mRNAs are rapidly degraded, followed by the ubiquitin-mediated proteolysis of SLBP. Interestingly, stabilizing SLBP does not prevent histone mRNA degradation, underscoring that multiple layers of regulation ensure the abrupt activation and termination of histone gene expression necessary to maintain histone supply during DNA replication [63,72].

In contrast with the canonical histones, the expression of replication-independent histones is more typical in terms of eukaryotic transcription. For example, genes encoding histone variants are not clustered and are instead distributed throughout the genome. The replication-independent genes have also been renamed using the new Strasbourg nomenclature, and their symbols denote the encoded variant and the core histone from which it stems [65]. Histone variants are also expressed throughout the entire cell cycle, and transcript stability does not rely on the expression of SLBP. Therefore, replication-independent histone mRNAs undergo routine splicing of their introns and are polyadenylated. Histone variant transcription is instead governed by cellular signals directing pathways such as DNA damage repair or development [73-78]. As a result, the expression of histone variants produces a supply of ‘replacement histones’ in times when chromatin and the epigenetic landscape must be dynamically remodeled.

### Synthesis of new histones

The next step in histone supply is the translation of the mRNA transcripts in the cytoplasm. Here, due to the histone’s net positive charge, a complex network of proteins co-operates to assist in histone folding, modification, and transportation back into the nucleus (Figure 1). This network includes heat shock proteins that aid in histone folding, enzymes that add PTMs to distinguish newly synthesized histones from recycled ones, and chaperones that transport and deliver the new histones to the nucleus [25,29,35,79-81]. Along with additional maturation in the cytoplasm, this process yields histones ready to be deposited into newly replicated DNA. Much like transcriptional regulation, the precise control of histone translation is essential to maintaining an adequate histone supply for chromatin assembly.

This process begins with the translation of H3 and H4 by free ribosomes. To prevent aggregation of the histones via their high net charge, heat shock cognate protein 70 (HSC70) and heat shock protein 70/90 (HSP90/HSP70) bind to the ribosome and assist with the proper folding of H3 and H4, respectively [29,35]. Histone H3 then undergoes its first modification with the addition of H3K9me1, which is placed co-translationally by the enzyme SETDB1 [82]. Translated H4 also interacts with the SET/PP32 complex which inhibits the histone’s premature acetylation. Disruption of this interaction and abnormal acetylation of H4 obstructs its interaction with HSP90, resulting in aberrant aggregation of the histone [36]. Next, the chaperones testis nuclear autoantigenic sperm protein (tNASP) and HSP90 aid in the heterodimerization of histones H3/H4 through interaction with their histone fold domains [29,35]. Histone chaperones somatic NASP, ASF1a, and retinoblastoma-binding protein p46 (RbAp46) then guide the acetylation of histone 4 at lysine 5 and 12 (H4K5K12ac) established by the enzyme histone acetyltransferase 1, marking the H3/H4 heterodimer as newly synthesized [29]. This modification persists until the heterodimer is incorporated into the DNA but is promptly removed after chromatin assembly [83]. A few additional modifications may be added to prepare the histone for its future role in transcriptional regulation or unknown functions (see below, Modifications of newly synthesized histones-a burgeoning field). Finally, ASF1-bound H3/H4 interacts with importin 4 to facilitate its translocation through the nuclear pore and into the nucleus [29,35,84].

Although the pathway and players involved in H3/H4 histone synthesis have been defined, more investigation is needed to completely understand this process. For example, one study proposes that different translocation pathways exist through distinct importin complexes [35]. These results suggest that translocation routes are differentiated according to the newly synthesized histone’s PTMs. In another study, it has been shown that histones H3 and H4 may translocate to the nucleus as monomers before heterodimerization within the nucleus [85]. Importantly, even less is known about the synthesis and maturation of the H2A/H2B dimer. Thus, more research is needed to clarify the details of the histone synthesis pathway to determine how the cell maintains a suitable supply of new histones for chromatin assembly, maintenance, and remodeling.

### Modifications of newly synthesized histones—a burgeoning field

In addition to H3K9me1 and H4K5K12ac mentioned above, newly synthesized H3/H4 heterodimers can undergo further modification in the cytoplasm [86,87]. These ‘extra’ PTMs remain poorly understood but may prime a subset of histones for incorporation into specific regions within the genome, increase that histone’s affinity for a binding partner, and regulate histone synthesis. Modifications including acetylation, methylation, and poly(ADP-ribosyl)ation of newly synthesized histones are therefore of interest to grasp whether their modification within the cytoplasm is necessary for sustaining chromatin structure and cellular identity.

Newly synthesized H3 can be acetylated at the residues K14 and K18 (H3K14K18ac) before its incorporation into chromatin. Although this modification is usually associated with actively transcribed regions of chromatin, the role of this modification pre-deposition into DNA is unknown. Interestingly, the histone variant H3.3, which is also associated with active transcription, is hyperacetylated in comparison with H3.1 [87]. The negative cross-talk of this modification with H3K9me1 has led groups to suggest that the early addition of H3K14K18ac and the deposition of these newly synthesized histones into chromatin may prime regions for transcriptional activation. Accordingly, the supply of H3K14K18ac-modified H3/H4 heterodimers may be necessary for changes in cellular identity during lineage priming or differentiation.

Opposed to acetylation, methylation of newly synthesized H3 histones in the cytoplasm is limited to H3K9me1 mentioned above. Approximately 30% of cytosolic histone H3 is observed with H3K9me1, which is a precursor to H3K9me3 [87]. Since H3K9me3 marks regions of heterochromatin, this suggests that early methylation of newly synthesized H3 prepares a subset of histones for deposition into transcriptionally repressed regions. Once incorporated into heterochromatin, H3K9me1 is converted to H3K9me2/3 by the enzyme SUV39H1/H2 [88,89]. Efficient reestablishment of these domains and their subsequent compaction is key to safeguarding the transcriptional repression of regions like DNA repeats and endogenous retroviruses during replication. Nevertheless, it cannot be ruled out that the H3K9me1 modification simply regulates the translation of H3 as SETDB1 (which deposits the modification) is associated with the ribosome during synthesis [82]. Thus, more studies regarding H3K9me1 are needed to deduce its role in histone synthesis and heterochromatin reformation.

Histones H3 and H4 can also acquire the less-studied modification, poly(ADP-ribosyl)ation during maturation. Loyola’s group has revealed that one or multiple ADP-ribose polymers may be added to individual histones bound with heat shock chaperone complexes [35]. Shortly after, the modification appears to be removed when H3 and H4 form the heterodimer. The role of this modification during histone maturation within the cytoplasm remains unclear, as well as the specific enzymes responsible for its addition and removal. The lack of antibodies or inhibitors for poly(ADP-ribosyl)ation has made the modification difficult to study, yet in recent years, several laboratories have developed different approaches to overcome this limitation [90-93]. These groups have shown that poly(ADP-ribosyl)ated histones are also found within chromatin and play an important role in regulating cellular processes such as DNA repair, replication, and transcription [94-97]. In the future, it will be essential to investigate poly(ADP-ribosyl)ated histones, which clearly play key roles within chromatin dynamics, so that we may better understand if this modification is important for the synthesis of H3 and H4.

## Deposition, storage, and degradation of histones

Finally, histones funneled in from the supply chain above must be deposited into DNA, stored for future use, or degraded (Figure 1). Particularly, a delicate balance between the deposition of newly synthesized histones and the recycling of parental histones must exist to redefine cellular expression patterns and identity after division. This is because newly synthesized histones (which are relatively unmodified) dilute the parental histones (which retain their PTMs) by two [15-20]. At this moment, the cell must either re-establish chromatin domains to match the mother cell epigenetic landscape or modulate chromatin domains to establish a new-found pattern. In either case, a precise supply of new and recycled histones is essential for establishing the correct gene expression profile in the resulting daughter cells.

### Newly synthesized histones

After maturation and entry into the nucleus, newly synthesized histones must be correctly placed into the DNA to replenish the reduced chromatin landscape (Figure 1). The histone chaperone ASF1 still accompanies the H3/H4 heterodimer in the nucleus until it hands the histone pair to another histone chaperone. In the case of the heterodimers containing the canonical histones H3.1 or H3.2/H4, that chaperone is the CAF-1 complex. Interestingly, in *Schizosaccharomyces pombe*, histone binding by the CAF-1 subunit, proliferating cell factor 1 (Pcf1), induces the folding of an IDR into an ordered structure. Cross-talk between this new protein fold and other IDRs within Pcf1 then mediates the interaction of CAF-1 with PCNA (the sliding clamp, proliferating cell nuclear antigen) and DNA at the replication fork [98]. The H3/H4 heterodimer is then deposited in pairs of two to yield a (H3/H4)_2_ tetramer partially wrapped in DNA [99]. Subsequently, the histone chaperone nucleosome assembly protein-1 (NAP-1) delivers two H2A/H2B dimers, resulting in a completed nucleosome [100]. Now in place, the newly established nucleosome must be modified to match its new chromatin environment.

PTMs deposited onto the newly entrenched histone are decided by the local surroundings. Often, neighboring, recycled histones constitute the desired domain and dictate the PTM placed on the newly synthesized histones within that region. For H3K27me3 or H3K9me3, the enzymatic polycomb repressive complex 2 (PRC2) complex and SUV39H1/H2 enzymes read the modifications of parental histones and write that modification onto the adjacent, new histones, respectively [88,101]. In actively transcribed regions, histone acetyltransferases and complexes such as COMPASS are recruited by RNA POLII, which is enriched at gene promoters [102]. As active domains are usually replicated first during S-phase, regions with modifications such as H3K4me3 are restored sooner than their repressed, H3K27me3 counterparts that undergo slow recovery well into G2/M phase [103,104]. Importantly, this delayed recovery of H3K27me3 domains may allow for the binding of transcription factors that mediate transcriptional activation and chromatin remodeling of certain loci during cellular differentiation. Therefore, the dynamics of re-establishing these domains are extremely important in safeguarding or modulating cellular identity before the next cellular division.

### Segregation of parental histones during DNA replication

An important stock of parental histones is recycled back into the DNA during replication and lays the groundwork for re-constituting the epigenome after cellular division (Figure 1). Ahead of the fork, the DNA helicase begins the disruption of pre-existing nucleosomes. Specifically, the subunit minichromosome maintenance complex component 2 (MCM2) disrupts the nucleosome with the help of facilitates chromatin transcription (FACT) and breaks it apart into H2A/H2B dimers and (H3/H4)_2_ tetramers [105,106]. The resulting tetramers are then passed to either the leading or lagging strand DNA polymerases, which help to incorporate them back into the DNA [46,48]. In the case of the lagging strand, MCM2 utilizes a histone-binding domain containing two critical tryptophan residues that mimic the aromatic bases found in DNA to bind the (H3/H4)_2_ tetramer and pass it to the lagging strand polymerase, Polα [46,50]. It is unclear whether FACT then re-deposits H2A/H2B dimers, reforming the nucleosome alone, or if another chaperone is involved. Although these are the well-defined players of histone recycling at the fork, recent studies have revealed that the replication checkpoint activation component mannose receptor, C Type 1-like (MRC1/CLASPIN) also functions as a histone chaperone critical for epigenetic inheritance. MRC1 binds H3-H4 tetramers alone or with MCM2 and facilitates their symmetrical segregation to daughter strands, thereby supporting heterochromatin maintenance [54,55,107]. Nevertheless, recycling parental histones yields an opportunity to transmit the epigenetic information to the daughter strands of DNA.

 The relaying of this epigenetic information relies on the correct repositioning of parental histones containing particular modifications. Key studies have shown the local recycling of the repressive, facultative heterochromatin modification, H3K27me3, which is retained and placed back into the same loci after DNA replication [108,109]. This retention relies on the specific histone chaperone NPM1 [56], whose observation is in line with the recycling of H3K9me histones by the subcomplex of HP1α-CAF-1-SETDB1 [89] and the specific recycling of CENPA by the histone chaperone HJURP, which interacts with MCM2 at centromeres [51]. Not long ago, it was thought that pre-existing H2A/H2B dimers were displaced and had little role in epigenetic memory. However, new studies are showing that the ubiquitination found in parental H2A/H2B dimers helps accurately restore stable H3-H4 chromatin states [110]. Of note, the specific retention of ubiquitination of lysine 119 of H2A (H2AK119ub), placed by the PRC1 complex, is epigenetically preserved [110]. Along with the placement of the parental nucleosome itself, the local environment around deposition is also important for the retention of epigenetic memory. For example, histone deacetylases actively stabilize nucleosomes carrying H3K9me3 to support heterochromatin propagation [111]. Whether modifications decorating active transcription are locally inherited is still debated, as histone turnover, histone variants, and the presence of HATs can muddy the methods used to assess the precise recycling of parental histones. Importantly, the faithful replacement of these parental histone modifications redefines chromatin domains and gene expression profiles, which ultimately affect cellular identity. Ergo, more studies should be performed to discover whether similar mechanisms of local retention exist for other histone modifications or variants.

### Storage and degradation of histone proteins

As part of their tightly regulated life cycle, histones undergo both storage and degradation to maintain genome integrity and ensure proper chromatin assembly (Figure 1). Histone storage has been extensively studied in the context of early development, particularly in oocytes, where maternally derived histones, bound to specific chaperones, are stored in the cytoplasm for use during the rapid, transcriptionally silent cell cycles that follow fertilization [112-114]. In *Drosophila* eggs, histones H2A and H2B are sequestered in lipid droplets through a mechanism dependent on the anchoring protein Jabba [115-117], while in mouse oocytes, H3 and H4 have also been found in lipid droplets [118]. Despite these insights, histone storage mechanisms outside of developmental contexts remain poorly understood. In somatic cells, newly synthesized H3.1 can be incorporated at DNA repair sites outside of S-phase in a CAF-1-dependent manner, suggesting that chromatin restoration involves not only parental histone recycling but also the availability of a regulated pool of soluble histones [119]. This highlights the existence of non-developmental histone storage mechanisms that warrant further exploration.

Alongside storage, histone degradation plays a crucial role in maintaining proper histone protein levels, as both histone insufficiency and excess can be detrimental to the cell [120]. An overabundance of histones can lead to nonspecific binding to DNA and RNA, disrupt chromatin organization, and alter gene expression [121]. To counteract this, cells have evolved multiple mechanisms that regulate histone levels and prevent their accumulation, particularly when DNA replication is blocked. These regulatory pathways include histone chaperones such as Asf1 to help buffer excess histones [122], while targeted histone degradation acts as a post-translational control mechanism [120]. In yeast, excess soluble histones are degraded by the proteasome in a Rad53-dependent manner, ensuring that unbound histones do not accumulate [120,123]. This degradation is signaled by the phosphorylation of histone H3 at tyrosine 99 (H3Y99ph) placed discreetly on non-chromatin-bound H3 [120]. In higher eukaryotes, histone degradation is similarly regulated through various PTMs. For example, excess histone H3 is phosphorylated at threonine 118 (H3T118ph) during mitosis, which directs the histone to centrosomes, where it undergoes ubiquitination followed by proteasome-mediated degradation [124]. Histones H3 and H4 may also be degraded in response to UV-induced DNA damage after being ubiquitinated by the cullin 4, DNA-binding protein 1, regulator of cullins-1 (CUL4-DDB-ROC1) ubiquitin ligase. The targeted removal of these histones then facilitates DNA repair of the damaged region [125].

The strict balance of histone storage and degradation is exemplified by the dynamic pool of soluble H3/H4 histones in mammals. The histone chaperone NASP stabilizes a portion of this histone pool, while the chaperone HSC70 targets excess histones for lysosomal degradation, a process known as chaperone-mediated autophagy (CMA). Under normal conditions, NASP and HSC70 compete for binding to H3 with a bias toward NASP, ensuring that histones are protected from degradation. However, under conditions where H3 begins to accumulate, the balance may shift in favor of HSC70 to promote CMA-dependent degradation. The dynamic interplay between these chaperones ensures that the cell maintains an adequate reservoir of H3/H4 to safeguard the chromatin landscape against threats like replication stress without exceeding the cell’s histone storage limit [126,127]. Beyond maintaining proper histone levels, CMA also functions as a folding checkpoint. Newly synthesized histones undergo a quality control step, and if proper folding is not achieved, they are degraded in a CMA-dependent manner [127]. These mechanisms collectively ensure that histone levels remain finely tuned, preventing genomic instability and protecting cellular identity.

## Consequences of disturbing the histone supply chain

All aspects of the histone supply chain, including histone gene expression, mRNA processing, protein synthesis, deposition, and recycling must function correctly to maintain epigenetic information and cellular identity. Challenges, particularly mutations to proteins involved in this process, often result in erroneous cellular differentiation or cancer due to aberrant chromatin structure and gene expression. Throughout the remainder of this review, we will explore and highlight examples where the histone supply chain has been disrupted and the impact of these complications on cellular identity.

Alterations to histone gene expression can significantly affect the progression of cancer. Surprisingly, it had not been shown until recently that cancer cells have increased expression of histones despite the high demand for them to accommodate accelerated cellular growth rates. Indeed, in a recent study, cleavage under targeted accessible chromatin (CUTAQ) was used to show that increased levels of RNA POLII are found at histone genes in cancer cells. This RNA POLII occupancy correlates with cancer aggressiveness [128] and presumably helps drive cancer progression by supplying these cells with ample chromatin-building material needed to proliferate uncontrollably. Likewise, impaired function of the upstream transcription factor NPAT has also been demonstrated in a variety of aggressive cancers, including Hodgkin’s lymphoma, colorectal cancers, and breast cancers [129-131]. In the case of breast cancer, NPAT is found to associate highly with CDK2, which permits the overexpression of canonical histones during and outside of S-phase [131]. Notably, the NPAT-CDK2 axis is also sensitive to DNA damage. For example, in response to genotoxic stress, transcription of histone genes is immediately repressed by disrupting NPAT phosphorylation and its association with histone gene clusters to limit histone overproduction during cell cycle arrest [132]. In any case, disrupting the first step in the histone supply chain and the expression of canonical histones may provide cancer cells with the means needed to proliferate and progress toward resilient malignancies.

The histone supply chain may also be impaired at the transcript level as disrupting the SLBP or the processing of mRNAs imbalances the histone pool and ultimately affects chromatin structure. Recent studies have found that histone mRNAs become aberrantly polyadenylated when SLBP is depleted [133]. As a result, H3.1 levels increase considerably and evict H3.3 variants from the chromatin, which alters cellular expression patterns toward malignant programs [134,135]. Known environmental carcinogens are also thought to act through this mechanism, as both nicotine and arsenic decrease cellular SLBP levels [133,136]. Beyond transcription, the maturation of histone mRNAs may also be disrupted; a recent study implicates a novel function of the histone chaperone ASF1 in regulating the 3′ UTR processing of canonical histone pre-mRNAs. Upon the chaperone’s depletion, there is a decrease in mature mRNA and histone protein levels while histone pre-mRNAs increase [137]. The authors suggest that ASF1 acts as a ‘chaperone checkpoint’ and that its expression helps to fine-tune histone supply during S-phase [137]. When this mechanism is left unchecked, such as in cancer cells with increased ASF1 expression, the uncontrolled supply of histones may feed the cells’ high demands during division. These studies demonstrate the necessity for precise and proper processing of histone mRNAs as these transcripts feed directly into the downstream protein supply chain.

Likewise, disrupting the maturation of newly synthesized histones may also imbalance the histone pool and affect chromatin structure. For instance, depletion of the enzyme jumonji domain-containing protein 1B (JMJD1B) increases cytoplasmic levels of the chaperone tNASP, which sequesters histones H3 and H4, preventing their nuclear import [138,139]. This results in histone scarcity within the nucleus and drives S-phase arrest, DNA damage, and chromosomal instability. As previously mentioned, the histone maturation process may also be disrupted by a reduction in SET/PP32 proteins. This induces the abnormal acetylation of H4 and impairs the histone’s interaction with HSP90, leading to aberrant aggregation in the cytoplasm [36]. Intriguingly, the histone chaperone DnaJ heat shock protein family member C9 (DNAJC9) may assist with both the initial folding of H3 and H4 histones and the resolution of their misfolding, as it can be found associated with HSP70 in the nucleus. This not only reveals a novel multifunctional co-chaperoning ability of DNAJC9 but also suggests its importance in regulating the downstream pool of H3/H4. Importantly, these histones feed into the ASF1-dependent supply pathway at various stages, and disruption of this co-ordination can result in improper histone loading and compromised chromatin integrity [140,141]. Future functional studies with separation of function mutants will be interesting to see if histone folding in the absence of this interaction also affects overall chromatin structure and cellular identity.

Once matured, it is imperative that each histone be transported and placed into the chromatin at the correct location by histone chaperones. Numerous groups have explored the ramifications of mutating or depleting key histone chaperones. When chaperones involved in *de novo* histone deposition, such as DNAJC9 or CHAF1-B (Chromatin Assembly Factor 1B, a subunit of CAF-1), are depleted, the cell suffers a severe shortage of canonical histones, which consequently permits the deviant placement of centromeric CENP-A histones to positions away from the centromere [142,143]. This leads to the misalignment of kinetochore machinery and chromosome instability, a hallmark of aggressive, solid tumors [144]. Conversely, disrupting the supply of histones themselves can directly affect histone chaperoning mechanisms. For example, when CENP-A is overexpressed, the histone variant is incorrectly carried and misplaced in the DNA by the histone chaperone DAXX, which is the typical chaperone of H3.3 [145]. Likewise, in breast cancer cells, metastatic stimulants suppress the CAF-1 complex and prevent the delivery of H3.1 and H3.2. In the absence of these core histones, the chaperone HIRA ‘gap-fills’ the naked DNA with H3.3. This shift remodels chromatin to favor the expression of poor-prognosis genes and EMT (epithelial-to-mesenchymal)-inducing transcription factors, driving tumorigenesis and metastasis, and linking altered histone variant usage to aggressive phenotypes [146].

Another striking effect occurs by disrupting the chaperoning activities of either MCM2 or POLE*ε* (DNA polymerase epsilon, catalytic subunit) in mouse embryonic stem cells (mESCs). In these mutant cells, recycling and transmission of parental histones to either the leading or lagging daughter strands of DNA is hindered [47]. The epigenetic information encoded by these recycled histones is thereby skewed to only one of the resulting daughter cells [147]. Large-scale effects of this attenuated mechanism appear when differentiating these mESCs toward neural progenitor cells as stem cell genes are not silenced, nor are lineage-specific genes activated [148]. Interestingly, simultaneously disrupting MCM2 and POLE chaperoning activity releases parental histones into a soluble pool, sequestering their important genetic information [149]. A final example is the high mutation rate of the specialized H3K27me3 chaperone NPM1 [56], which is mutated in 30% of AML cases [150]. Disease cells with altered NPM1 show de-repression of key developmental genes, including the Hox loci [151]. However, how the chaperone’s mechanism or the histone supply is disrupted in these cases remains unknown. NPM1 is part of a ‘laundry list’ of histone chaperones that can be misregulated in different cancers—a subject reviewed elsewhere by multiple groups [22,59,152]. Overall, these case studies emphasize the crucial role of histone chaperones in the histone supply chain and in maintaining cellular identity.

## Conclusions

Together, we have summarized key aspects of the histone supply chain, including the protein players involved in histone expression, transcription, protein synthesis, movement, and deposition into chromatin. Although many steps of these processes are defined, many details remain unknown. In years to come, we expect groups to continue characterizing histones' impact on chromatin environments and the chaperones and enzymes that mediate them. This will be especially true as structural techniques such as cryo-EM and protein structure prediction tools continue to improve so that we may understand how molecular structure affects chromatin dynamics. We also anticipate the deconvolution of poorly understood mechanisms such as H2A/H2B synthesis and locomotion, transient modification during histone synthesis, identification of specialized histone chaperones, and the epigenetic inheritance of active chromatin domains. Undoubtedly, high-resolution tools will be needed to account for the rapid pace of these systems and the sheer number of potential proteins involved. We have seen the birth of these tools in recent years with single-cell RNA-sequencing [150] and proteomics [151], as well as cleavage under targets and tagmentation (CUT&Tag) [152]. With this knowledge, we will better understand how each piece of the histone supply chain affects epigenetic memory and cellular identity.

With a clearer picture of the histone supply chain, we will increase our capacity to treat human diseases. For example, understanding the structure-guided mechanism of a particular histone chaperone will help determine the impact of when that mechanism is impaired. It will also help design therapeutics for diseases implicated in the aberrant function of a mutated protein. With the advent of AI (artificial intelligence) and *de novo* protein design [153], we can also expand our capabilities to manipulate these mechanisms for optimal histone supply and epigenetic inheritance. Thus, comprehending the impact of each contributor to histone dynamics and supply will, in turn, improve vital treatments in cancer, stem cell therapies, and regenerative medicine.

## Abbreviations

CAF-1: chromatin assembly factor-1
CENP-A: centromere protein A
CMA: chaperone-mediated autophagy
DNAJC9: DnaJ heat shock protein family member C9
HJURP: Holliday junction recognition protein
HLB: histone locus bodies
HP1: heterochromatin protein 1
HSC70: heat shock cognate protein 70
IDRs: intrinsically disordered domains
NPM1: nucleophosmin
PHD: plant homeodomain finger
PHF8: PHD finger protein 8
PTMs: post-translational modifications
SETDB1: SET domain bifurcated histone lysine methyltransferase 1
SLBP: stem-loop binding protein
SUV39H1/H2: suppressor of variegation 3-9 homolog 1/2
asf1: anti-silencing function
tNASP: testis nuclear autoantigentic sperm protein
